# CXCL5 promotes gastric cancer metastasis by inducing epithelial-mesenchymal transition and activating neutrophils

**DOI:** 10.1038/s41389-020-00249-z

**Published:** 2020-07-06

**Authors:** Zheying Mao, Jiahui Zhang, Yinghong Shi, Wei Li, Hui Shi, Runbi Ji, Fei Mao, Hui Qian, Wenrong Xu, Xu Zhang

**Affiliations:** 1 Jiangsu Key Laboratory of Medical Science and Laboratory Medicine, School of Medicine, Jiangsu University, Zhenjiang, Jiangsu 212013 China; 2 Center of Research Laboratory, First People’s Hospital of Lianyungang, Lianyungang, Jiangsu 222001 China; 3 Department of Clinical Laboratory Medicine, The Affiliated People’s Hospital of Jiangsu University, Zhenjiang, Jiangsu 212002 China

**Keywords:** Gastric cancer, Inflammation, Gastric cancer, Inflammation

## Abstract

Deregulated expression of chemokines in tumor microenvironment contributes to tumor metastasis by targeting distinct cells. Epithelial-derived neutrophil-activating peptide-78 (ENA78/CXCL5) is upregulated in many cancers and involved in tumor progression. The role and underlying mechanism of CXCL5 in gastric cancer (GC) metastasis remain unclear. In this study, we reported that the expression of CXCL5 was elevated in tumor tissues and positively associated with lymphatic metastasis and tumor differentiation. Stimulation by recombinant human CXCL5 (rhCXCL5) induced epithelial-mesenchymal transition (EMT) in GC cells through the activation of ERK pathway, which enhanced their migration and invasion abilities. The culture supernatant from tumor tissues also enhanced the migration and invasion abilities of GC cells, however, this effect was reversed by pre-treatment with CXCL5 neutralizing antibody. Further studies showed that rhCXCL5 could induce the expression of IL-6 and IL-23 in neutrophils through the activation of ERK and p38 signaling pathways, which in turn facilitated GC cell migration and invasion. The culture supernatant from tumor tissues showed similar effects on neutrophils in a CXCL5-dependent manner. Blockade of IL-6 and IL-23 with neutralizing antibodies reversed the induction of EMT and the increased migration and invasion abilities in GC cells by CXCL5-activated neutrophils. Moreover, CXCL5 activated neutrophils could promote gastric cancer metastasis in vivo. Taken together, our results indicate that CXCL5 acts on gastric cancer cells to induce EMT and mediates pro-tumor activation of neutrophils, which synergistically promotes the metastatic ability of GC cells.

## Introduction

Gastric cancer (GC) is the fifth common cancer and ranks the third leading cause of cancer-associated deaths worldwide^1^. Although the incidence and mortality of GC appears to decrease globally, the prognosis of GC patients remains poor due to the high rate of metastasis and recurrence and limited effective treatments^2^. Inflammation is one of the hallmarks of cancer. Inflammatory microenvironment has been shown to be critically involved in the development and progression of gastric cancer^3^. Chemokines, a multifunctional family of small highly conserved proteins (8–12 kDa), controls many biological processes, especially leukocyte trafficking^4^. Chemokines have been found to be essential for cancer cell proliferation, migration and invasion^5^. Epithelial-mesenchymal transition (EMT) plays a key role in metastasis by enhancing plasticity, mobility, and invasion of cancer cells. Chemokines and its receptors are reported to regulate cancer development and progression through EMT^6^.

Chemokines expressed at tumor site are considered as principal factors that interact with immune cells to dictate tumor development and progression^7^. For instance, in triple-negative breast cancer, CXCL16 derived from cancer-associated fibroblasts attracts monocytes and amplifies stroma activation, which contributes to the aggressive phenotype^8^. In colorectal cancer, CXCL8 recruits neutrophils into tumor site, which inhibits local immunity and in turn contributes to tumor development^9^. Epithelial neutrophil-activating peptide-78 (ENA78/CXCL5), a member of the ELR^+^ CXC chemokine family, is identified as an inflammatory mediator with powerful role in neutrophil chemotaxis^10^. Overexpression of CXCL5 is found in various cancers and promotes tumor growth and metastasis^11,12^. CXCL5 increases the metastatic potential of breast cancer cells via upregulation of Snail^13^. In nasopharyngeal carcinoma, CXCL5 induces EMT through the activation of the ERK/GSK-3β/Snail signaling pathway, which promotes cell migration and invasion and lung metastases^14^. In addition, CXCL5 from cancer-associated fibroblasts increases PD-L1 expression in melanoma and colorectal cancer cells^15^. These studies suggest that CXCL5 plays diverse roles in tumor development and progression.

Over the past decades, a variety of studies suggest that tumor-associated neutrophils are involved in tumorigenesis, tumor growth, metastasis, angiogenesis, and immunosuppression^16^. Wang et al. suggest that tumor-associated neutrophils induce EMT in breast cancer cells through the CD90-TIMP-1 interaction loop^17^. Tumor-associated neutrophils derived factors such as VEGF and MMP9 contribute to tumor angiogenesis and metastasis^18^. Our previous study has shown that IL-6 could mediate the interaction between cancer-derived mesenchymal stem cells and neutrophils through STAT3 signaling pathway, which ultimately promotes gastric cancer cell migration and angiogenesis^19^. Zhou et al. demonstrate that overexpression of CXCL5 mediates neutrophil infiltration and indicates poor prognosis for hepatocellular carcinoma (HCC)^20^. Neutrophil infiltration mediated by CXCL5 in laryngeal squamous cell carcinoma microenvironment leads to tumor cells escaping immune surveillance^21^. Based on these studies, we hypothesized that CXCL5 may exert dual roles in both cancer cells and microenvironmental cells such as neutrophils, finally accelerating gastric cancer progression.

In the present study, we investigated the role of CXCL5 in gastric cancer metastasis and explored the underlying mechanism. Our findings showed that CXCL5 was upregulated in GC and its upregulation was associated with lymphatic metastasis and tumor differentiation. CXCL5 induced EMT in GC cells via regulation of ERK/Snail pathway and pro-tumor activation of neutrophils, which cooperatively promoted gastric cancer metastasis. Our findings suggest that CXCL5 exerts tumor-promoting roles in gastric cancer by acting on both cancer cells and neutrophils, suggesting that it may serve as a potential target for gastric cancer therapy.

## Results

### CXCL5 expression is upregulated in human gastric cancer

**Fig. 1:**
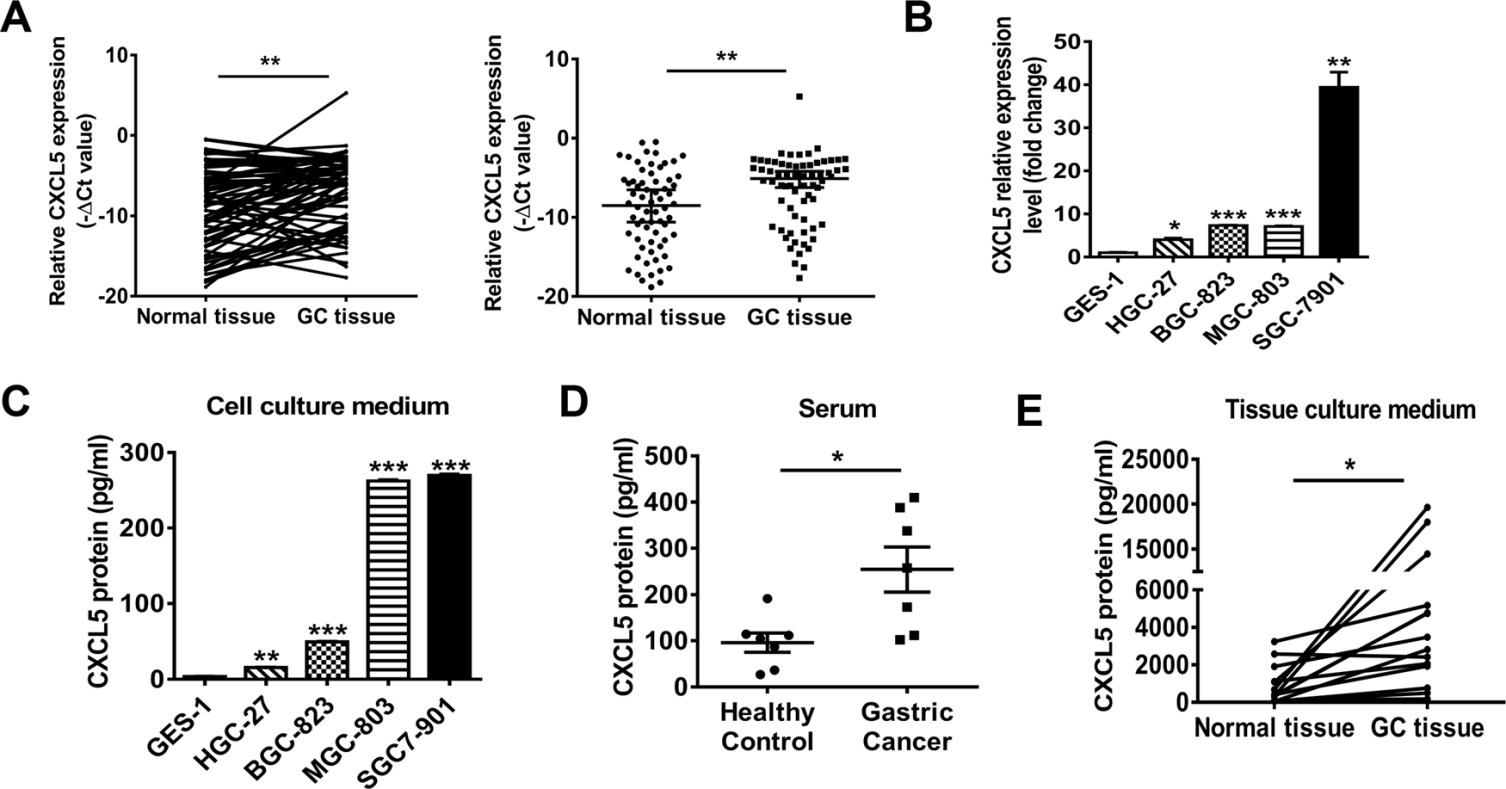
CXCL5 is upregulated in human gastric cancer. **a** QRT-PCR analyses of CXCL5 expression in gastric cancer tissues and non-tumor tissues. **b** QRT-PCR analyses of CXCL5 expression in gastric cancer cell lines and normal gastric epithelial cell line. **c** ELISA assays for CXCL5 concentrations in culture supernatants from gastric cancer cell lines and normal gastric mucosa epithelial cell line. **d** ELISA assays for CXCL5 concentrations in the serum of gastric cancer patients and healthy controls. **e** ELISA assays for CXCL5 concentrations in culture supernatants from tumor tissues and non-tumor tissues.

To investigate the role of CXCL5 in gastric cancer, we first examined the expression of CXCL5 in a total of 66 paired gastric tumor tissues and adjacent non-tumor tissues and four human GC cell lines. As shown in Fig. 1a, b, an elevated expression of CXCL5 was observed in tumor tissues and GC cell lines compared to non-tumor tissues and normal gastric epithelial cell line, respectively. High expression of CXCL5 was associated with lymphatic metastasis and tumor differentiation but had no relevance with age, gender, tumor size, and invasion depth (Table 1). The concentrations of CXCL5 in serum from GC patients, culture supernatants from tumor tissues and GC cell lines were also found to be higher than that from healthy controls, non-tumor tissues and normal gastric epithelial cell line (Fig. 1c–e). These data suggest that CXCL5 is overexpressed in gastric cancer and high level of CXCL5 is positively associated with cancer progression.

**Table 1:** The association between CXCL5 expression levels and clinicopathological factors in gastric cancer patients.

	Number	CXCL5 Expression		*P* value
		High	Low	
Gender				0.654046
Male	47	32	15	
Female	19	14	5	
Age				0.247970
≤60	16	13	3	
≥60	40	33	17	
Tumor size				0.908862
≥5 cm	37	26	11	
<5 cm	29	20	9	
Nodal classification				0.033248
N0	14	6	8	
N1–3	52	38	14	
Invasion depth				0.506001
T1–2	9	7	2	
T3–4	57	38	19	
TNM stage				0.086692
I/II	20	11	9	
III/IV	46	35	11	
Differentiation				0.037958
Moderate	24	13	11	
Poor	42	33	9	

### CXCL5 promotes the migration and invasion of gastric cancer cells by inducing EMT

**Fig. 2:**
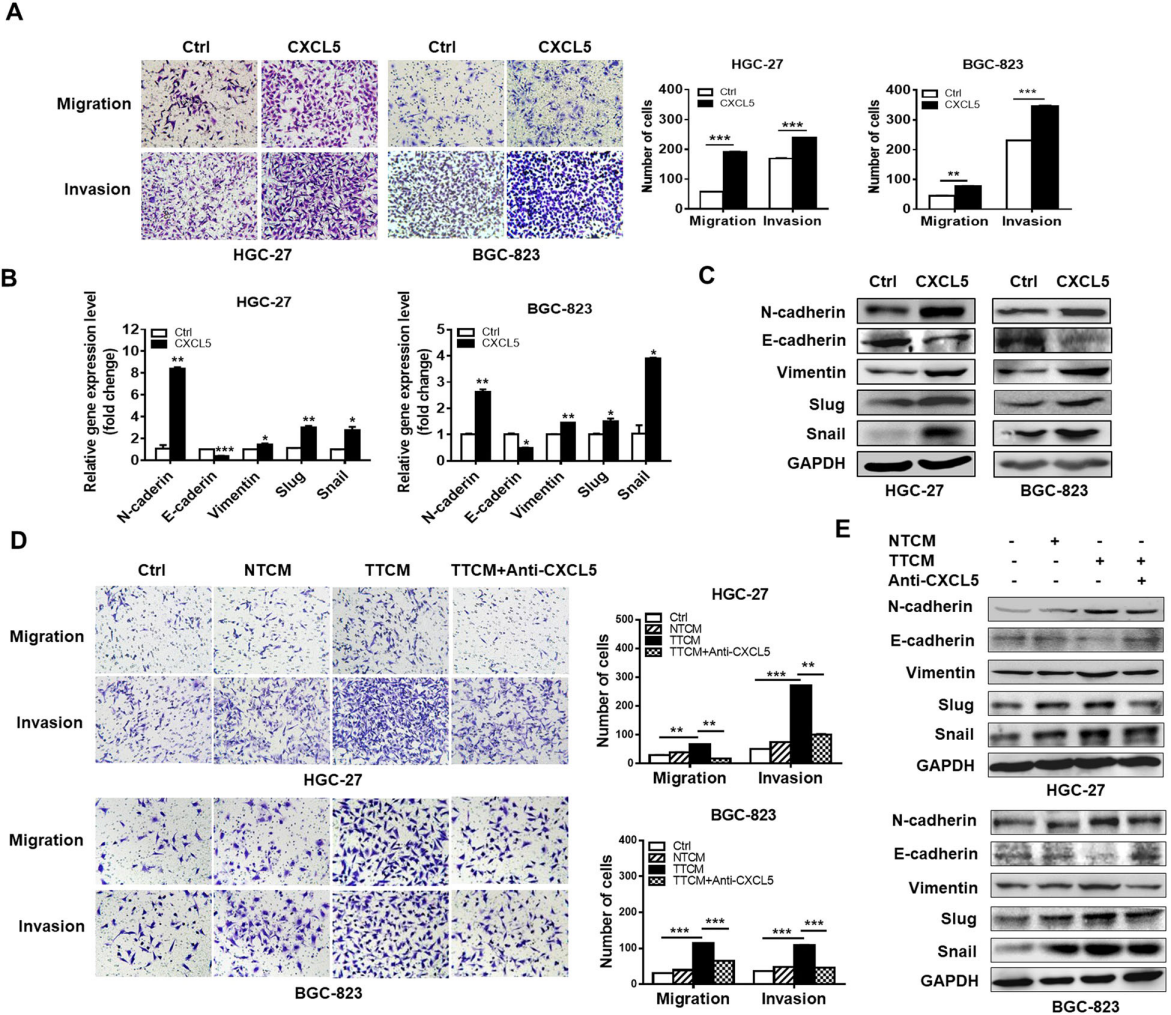
CXCL5 enhances GC cell migration and invasion by inducing EMT. **a** Transwell migration and Matrigel invasion assays for GC cells treated with or without CXCL5. **b**, **c** QRT-PCR (**b**) and western blot (**c**) analyses of the expression of EMT markers in GC cells treated with or without CXCL5. **d** Transwell migration and Matrigel invasion assays for GC cells treated with culture supernatants from tumor tissues (TTCM) and normal tissues (NTCM) in the presence or absence of CXCL5 neutralizing antibody. **e** Western blot assays for the expression of EMT markers in GC cells treated with TTCM in the presence or absence of CXCL5 neutralizing antibody.

To specifically address the importance of CXCL5 in GC metastasis, we performed transwell experiments to observe the effects of CXCL5 on the migration and invasion of GC cells. We chose HGC-27 and BGC-823 cells for further studies because they expressed relatively low levels of CXCL5 and were more sensitive to CXCL5 stimulation. We found that cells in rhCXCL5-treated groups showed an increased migration and invasion potential than control group (Fig. 2a). To test whether CXCL5 promotes gastric cancer cell migration and invasion by inducing EMT, we detected the expression of EMT markers in CXCL5-treated GC cells. The results of QRT-PCR and western blot showed that CXCL5 treatment decreased the expression of epithelial marker E-cadherin while increased that of mesenchymal markers N-cadherin and Vimentin in GC cells. In addition, the expression of EMT transcription factors Snail and Slug was also increased in CXCL5-treated GC cells (Fig. 2b, c). Since CXCL5 is highly expressed in the culture supernatants from tumor tissues, we incubated GC cells with tumor tissue culture medium (TTCM) and non-tumor tissue culture medium (NTCM) and found that cells incubated with TTCM also exhibited increased migration and invasion abilities compared to those incubated with NTCM. Moreover, the enhancement of cell migration and invasion by TTCM could be abrogated by CXCL5 neutralizing antibody (Fig. 2d). The results of western blot showed a similar change trend in the expression of EMT markers as that observed for rhCXCL5 treatment, which indicates that CXCL5 in gastric cancer microenvironment could induce EMT in GC cells (Fig. 2e). Taken together, these findings suggest that CXCL5 enhances the migration and invasion abilities of gastric cancer cells through the induction of EMT.

### CXCL5 activates ERK pathway to induce EMT in GC cells

**Fig. 3:**
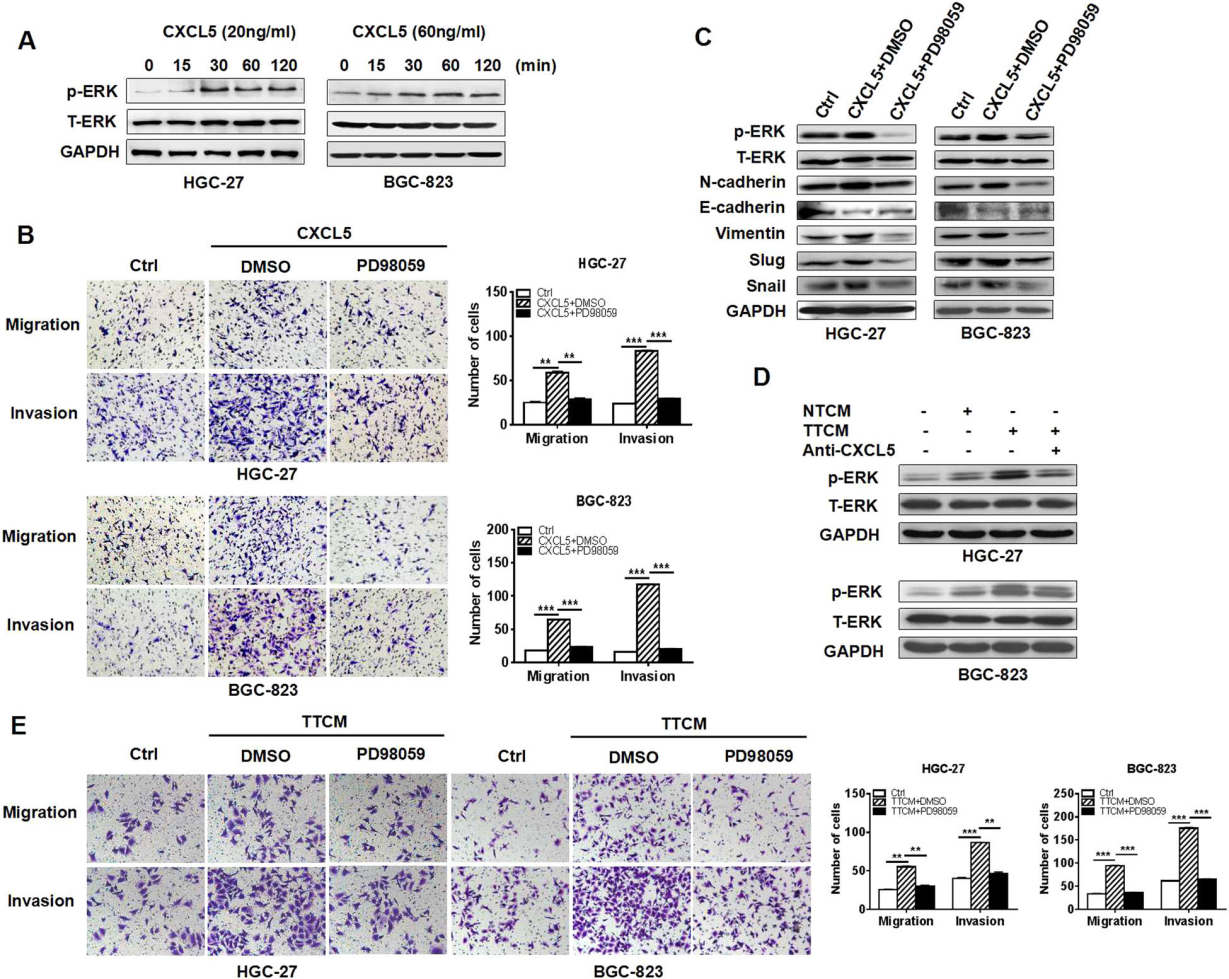
CXCL5 activates ERK signaling pathway to promote GC cell migration and invasion. **a** Western blot assays for the expression of phosphorylated ERK in GC cells treated with CXCL5 for different times. **b** Transwell migration and Matrigel invasion assays for GC cells treated with CXCL5 in the presence or absence of ERK inhibitor PD98059. **c** Western blot assays for the expression of EMT markers in GC cells treated with CXCL5 in the presence or absence of ERK inhibitor PD98059. **d** Western blot assays for the expression of phosphorylated ERKs in GC cells treated with TTCM in the presence or absence of CXCL5 neutralizing antibody. **e** Transwell migration and Matrigel invasion assays for GC cells treated with TTCM in the presence or absence of ERK inhibitor PD98059.

CXCL5 has been shown to activate ERK1/2 and PI3K/Akt signaling pathways in HCC cells by interacting with its receptor CXCR2^12^ and activate the ERK/GSK-3β/snail signaling pathway in nasopharyngeal carcinoma cells^14^. In colorectal cancer cells, ERK/GSK-3β/snail signaling pathway is also activated by CXCL5^22^. We found that CXCL5 stimulation upregulated the expression of phosphorylated ERK at 30–60 min after treatment (Fig. 3a). Additionally, PD98059, which specifically inhibits the phosphorylation of ERK, reversed the promotion of GC cell migration and invasion by CXCL5 (Fig. 3b). More importantly, PD98059 reduced the expression of N-cadherin, Vimentin, Snail, and Slug but increased that of E-cadherin in CXCL5-treated GC cells (Fig. 3c). Blockade of CXCL5 by neutralizing antibody inhibited the phosphorylation of ERK by TTCM in GC cells and inhibition of ERK decreased the promotion of GC cell migration and invasion by TTCM (Fig. 3d, e). In summary, these results suggest that ERK pathway is critically involved in CXCL5-mediated EMT in GC cells.

### CXCL5 induces neutrophil chemotaxis and activates neutrophil through ERK and p38 signaling pathways

**Fig. 4:**
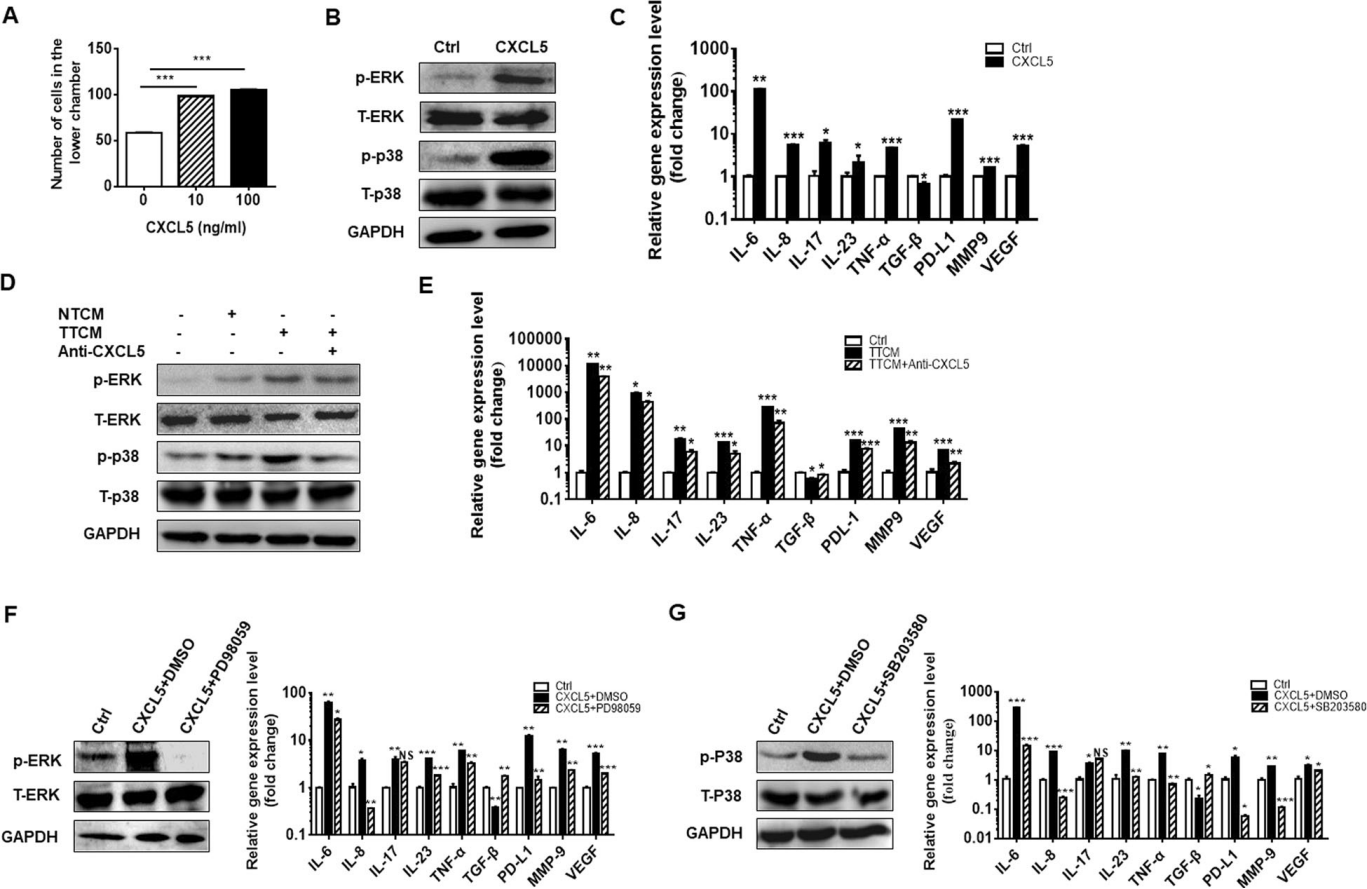
CXCL5 induces neutrophil chemotaxis and activates neutrophils through ERK and P38 signaling pathways. **a** Chemotaxis assay for neutrophils induced by different concentrations of CXCL5 (10 and 100 nM). **b** Western blot assays for the expression of phosphorylated ERK and P38 in neutrophils treated with CXCL5. **c** QRT-PCR analyses of the expression of IL-6, IL-8, IL-17, IL-23, TNF-α, TGF-β, MMP9, VEGF, and PD-L1 in neutrophils treated with CXCL5. **d** Western blot assays for the expression of phosphorylated ERK and P38 in neutrophils treated with TTCM in the presence of CXCL5 neutralizing antibody. **e** QRT-PCR analyses of the expression of IL-6, IL-8, IL-17, IL-23, TNF-α, TGF-β, MMP9, VEGF, and PD-L1 in neutrophils treated with TTCM in the presence of CXCL5 neutralizing antibody. **f**, **g** QRT-PCR analyses of the expression of IL-6, IL-8, IL-17, IL-23, TNF-α, TGF-β, MMP9, VEGF, and PD-L1 in CXCL5-treated neutrophils in the presence or absence of ERK inhibitor PD98059 (**f**) and p38 inhibitor SB203580 (**g**).

CXCL5 is a powerful attractant for neutrophils. We have previously shown that tumor-activated neutrophils promote gastric cancer cell migration and invasion by inducing EMT^23^. Hence, we hypothesized that CXCL5 might act on neutrophils to substantially influence GC metastasis. We first studied the direct effect of CXCL5 on neutrophils. We found that rhCXCL5 induced the chemotaxis of neutrophils in a dose-dependent manner (Fig. 4a). To understand whether CXCL5 could activate neutrophils, we performed western blot to detect the status of four signaling pathways involved in neutrophil activation, including ERK, p38, NF-kB, and STAT3, in neutrophils with or without rhCXCL5 treatment. The phosphorylation levels of ERK and p38 were significantly upregulated in CXCL5-treated neutrophils (Fig. 4b). No significant changes were observed for phosphorylated NF-kB and STAT3 in CXCL5-treated neutrophils. QRT-PCR results showed that the expression levels of IL-6, IL-8, IL-17, IL-23, TNF-α, MMP9, PD-L1, and VEGF were upregulated and that of TGF-β was down-regulated in neutrophils treated with rhCXCL5 (Fig. 4c). Similarly, neutrophils cultured with TTCM (containing high level of CXCL5) also showed increased expression of p-ERK and p-p38. The addition of anti-CXCL5 antibody into TTCM partially reversed the activation of ERK and p38 and the increased expression of inflammatory factors in neutrophils (Fig. 4d, e). On the contrary, PD98059 and SB203580, specific inhibitors for ERK and P38 signaling pathways, effectively inhibited the expression of these factors in neutrophils treated with rhCXCL5 and TTCM (Fig. 4f, g). Taken together, these results suggest that neutrophils are activated by CXCL5 through ERK and p38 pathways and secret more substances related to inflammation, angiogenesis, and immunosuppression.

### CXCL5-activated neutrophils promote gastric cancer cell migration and invasion through IL-6 and IL-23

**Fig. 5:**
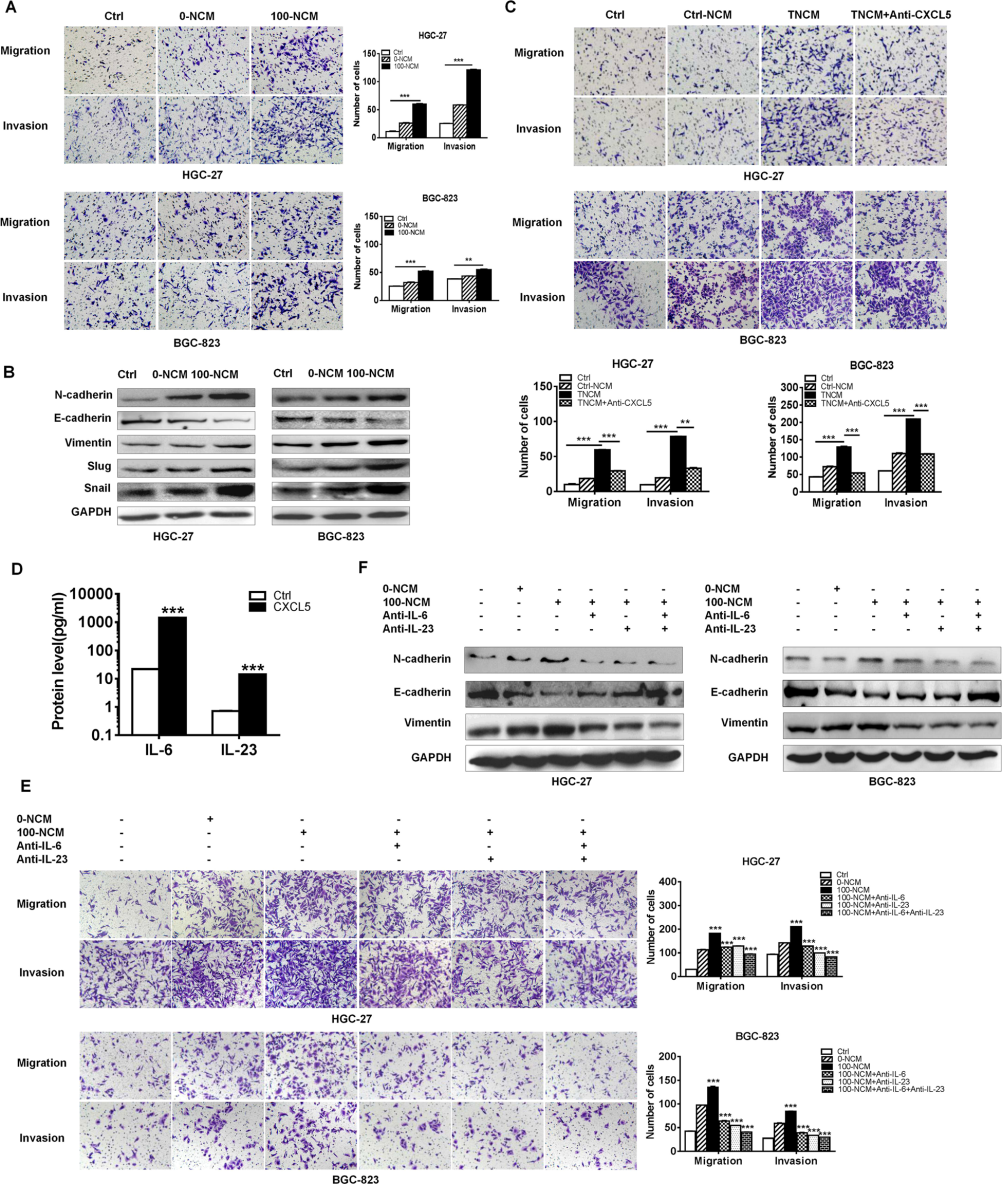
CXCL5-activated neutrophils promote GC cell migration and invasion by inducing EMT through IL-6 and IL-23. **a** Transwell migration and Matrigel invasion assays for GC cells treated with conditioned medium of untreated (0-NCM) and CXCL5 (100 nM)-treated (100-NCM) neutrophils. **b** Western blot assays for the expression of EMT markers in GC cells treated with conditioned medium of untreated and CXCL5-treated neutrophils. **c** Transwell migration and Matrigel invasion assays for GC cells treated with conditioned medium of untreated (NCM) or TTCM-primed neutrophils (TNCM) in the presence or absence of CXCL5 neutralizing antibody. **d** ELISA assays for IL-6 and IL-23 concentrations in culture supernatants from CXCL5-primed neutrophils. **e** Transwell migration and Matrigel invasion assays for GC cells treated with conditioned medium of CXCL5-activated neutrophils in the presence or absence of IL-6 and IL-23 neutralizing antibodies. **f** Western blot assays for the expression of EMT markers in GC cells treated with conditioned medium of CXCL5-activated neutrophils in the presence or absence of IL-6 and IL-23 neutralizing antibodies.

CXCL5 facilitates melanoma cell-neutrophil interaction and lymph node metastasis^24^. We further investigated the biological roles of CXCL5-activated neutrophils in GC progression. The results of cell migration and invasion assays showed that the conditioned medium of rhCXCL5 primed-neutrophils (100-NCM) apparently enhanced the migration and invasion abilities of GC cells while that of unprimed-neutrophils (0-NCM) had minimal effects (Fig. 5a). The results of western blot showed that the expression of N-cadherin, Vimentin, Snail, and Slug was upregulated in 100-NCM group while that of E-cadherin was down-regulated (Fig. 5b), which may explain the enhanced cell migration and invasion. We further used TTCM to activate neutrophils in the presence or absence of CXCL5 neutralizing antibody to see whether CXCL5 in gastric microenvironment boost tumor development by regulating neutrophils. As shown in Fig. 5c, the conditioned medium of TTCM-primed neutrophils (TNCM) also facilitated the migration and invasion of GC cells. While in the presence of CXCL5 neutralizing antibody, the promoting roles of TTCM-primed neutrophils were attenuated. We next investigated the specific factor by which CXCL5-primed neutrophils promoted GC cell migration and invasion. The previous studies have shown that IL-6 and IL-23 expression was significantly increased by CXCL5 treatment in neutrophils. ELISA results showed that the supernatants from CXCL5-primed neutrophils contained high levels of IL-6 and IL-23, which additionally justified the CXCL5-dependent secretion of IL-6 and IL-23 (Fig. 5d). The studies from other groups have shown that tumor-associated neutrophils induce EMT and promote gastric cancer cell migration and invasion^25^. To determine whether IL-6 and IL-23 secreted from CXCL5-activated neutrophils are responsible for the enhanced migration and invasion abilities of GC cells, we incubated GC cells with conditioned medium of CXCL5-primed neutrophils in the presence or absence of IL-6 and IL-23 neutralizing antibodies. As shown in Fig. 5e, the increased migration and invasion potential of GC cells was notably weakened in IL-6 and IL-23 blockade groups compared to control group. Western blot results showed that the expression of N-cadherin and Vimentin decreased while that of E-cadherin increased in 100-NCM-treated GC in the presence of IL-6 and IL-23 neutralizing antibodies (Fig. 5f). Therefore, these results clearly show the importance of IL-6 and IL-23 from CXCL5- activated neutrophils in promoting GC cell migration and invasion.

### CXCL5-activated neutrophils promote gastric cancer metastasis in vivo

**Fig. 6:**
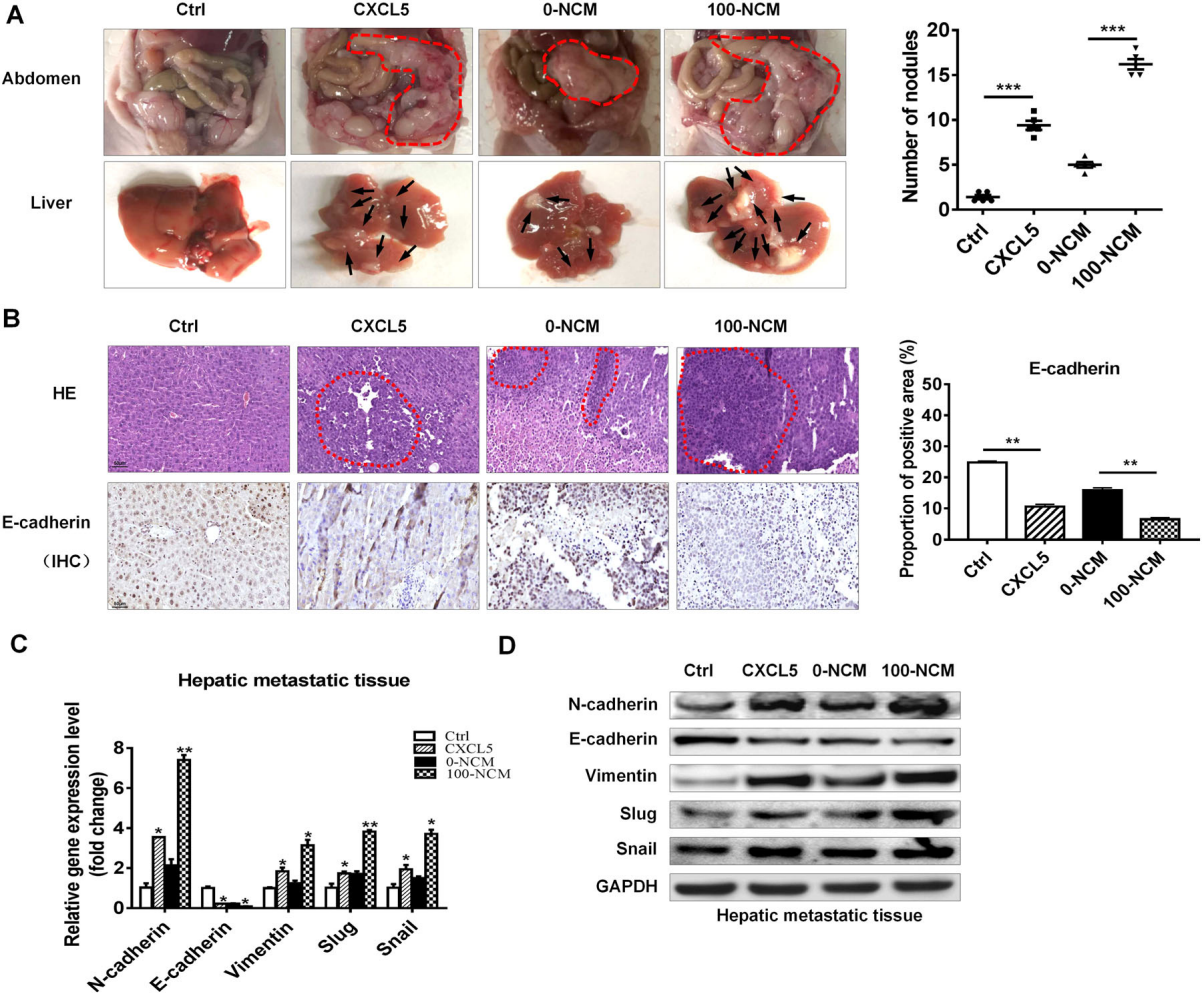
CXCL5 and CXCL5 primed-neutrophils promote gastric cancer metastasis. **a** The number of tumor colonized into the abdomen and the metastatic nodules in liver of mice in control, CXCL5, 0-NCM, and 100-NCM groups. **b**, **c** H&E staining and immunohistochemical staining of liver tissues for hepatic metastases and E-cadherin in mice from control, CXCL5, 0-NCM, and 100-NCM groups. **c**, **d** QRT-PCR (**c**) and western blot (**d**) analyses of the expression of EMT markers in mouse metastatic tumor nodules. Scale bar: 50 μm.

To further determine the effects of CXCL5-activated neutrophils on gastric cancer metastasis, we established a peritoneal metastasis model in nude mice. BGC-823 cells stimulated with or without rhCXCL5 and BGC-823 cells cultured with or without 100-NCM were injected into the abdominal cavity of nude mice. The metastatic tumor nodes in the abdomen were monitored for 3 weeks after injection. The results showed that more tumor nodules were colonized in the abdomen and more metastatic tumor nodules were observed in the livers of mice in CXCL5 and 100-NCM groups than that in control and 0-NCM groups, respectively (Fig. 6a). Hematoxylin and eosin staining results showed that there were more hepatic metastases in mice from CXCL5 and 100-NCM groups than those from control and 0-NCM groups, respectively. The results of immunohistochemical staining showed decreased expression of epithelial marker E-cadherin in CXCL5 and 100-NCM groups compared to control and 0-NCM groups, respectively (Fig. 6b). The results of QRT-PCR and western blot showed that the expression of N-cadherin, Vimentin, Snail, and Slug was increased, while that of E-cadherin was decreased in liver tissues in CXCL5 and 100-NCM groups compared to control and 0-NCM groups, respectively (Fig. 6c, d). These data indicate that CXCL5 and CXCL5-activated neutrophils promote gastric cancer metastasis in vivo.

## Discussion

Chemokines are directly implicated in a series of cellular functions, including cell proliferation, migration, and invasion^26^. Liu et al. demonstrate that CX3CL1 promotes lung cancer cell migration and invasion through the Src/FAK signaling pathway^27^. In intrahepatic cholangiocarcinoma, higher expression of CXCL12 is associated with metastasis and poor prognosis^28^. CXCL5 plays an important role in the development and progression of many cancers. Xu et al. demonstrate that CXCL5 promotes liver cancer migration and invasion^29^. Roca et al. show that apoptosis-induced CXCL5 accelerates inflammation and growth of prostate cancer metastasis in bone^30^. In lung cancer, Schwann cells secreted-CXCL5 is responsible for metastasis by inducing EMT^31^. In the current study, we found that the expression of CXCL5 was remarkably increased in GC tissues compared with adjacent non-cancerous tissues. In addition, overexpression of CXCL5 was associated with lymphatic metastasis and tumor differentiation. Stimulation with CXCL5 promoted the metastatic ability of GC cells in vitro and in vivo. We explored the mechanism for CXCL5-mediated metastasis and found that CXCL5 enhanced GC cell migration and invasion by activating ERK signaling pathway and inducing EMT. Notably, the culture supernatant from tumor tissues which contained a high concentration of CXCL5 also recapitulated these effects which could be abolished by CXCL5 neutralizing antibody. These findings suggest that CXCL5 may be a mediator and indicator of gastric cancer metastasis.

Chemokines interact with multiple types of cells in tumor microenvironment to exert diverse roles. Cancer-associated fibroblast-derived CXCL16 induces stroma activation and contributes to the aggressive phenotype of triple-negative breast cancers by acctracting monocytes^8^. Ogawa et al. have identified a CXCL1/8-CXCR2 axis in SMAD4 negative colorectal cancer, which promotes cancer progression by recruiting tumor-associated neutrophils^32^. CXCL8 derived from tumor-associated macrophages is associated with decreased CD8^+^ T cell infiltration and inhibits its function by inducing the expression of PD-L1 on macrophages, which indicates poor prognosis in gastric cancer patients^33^. CXCL5 is initially identified as a neutrophil-activating inflammatory peptide with homology to interleukin 8^34^. The previous studies in malignant diseases suggest that CXCL5 has a strong ability to recruit neutrophils. The recruitment of tumor-associated neutrophils in NSCLC involves SOX2-mediated production of CXCL5^35^. In addition, CXCL5 induced by TGF-β and Axl synergistically results in the infiltration of neutrophils into HCC tissue^36^. We showed that CXCL5 exhibited a strong chemotactic activity to neutrophils and enhanced the expression of several factors that have been reported to be responsible for their pro-tumor roles in neutrophils. CXCL5 activated ERK and p38 signaling pathways in neutrophils to induce their activation. More importantly, the increased phosphorylation of p38 and ERK in neutrophils by TTCM was abolished by CXCL5 blockade. Meanwhile, the elevated expression of pro-tumor factors in neutrophils by TTCM was also down-regulated when CXCL5 neutralizing antibody was added. Thus, CXCL5 may function as an efficient inducer of neutrophil activation in GC progression.

Neutrophils in tumor microenvironment possess anti-tumor and pro-tumor subtypes, which are classified according to their activation status and effects on tumor cells. Neutrophils directly interact with circulating tumor cells to support cell cycle progression in the circulation and to accelerate metastatic seeding^37^. Singhal et al. have identified an anti-tumor subset of tumor-associated neutrophils that function as antigen-presenting cells and augments anti-tumor effector T cell responses in early-stage human lung cancer^38^. Neutrophils are attracted to the tumor microenvironment by cytokines and chemokines, whereby neutrophils polarize to a pro-tumor subtype to promote tumor growth, metastasis, angiogenesis, and induce immunosuppression^39^. Zhou et al. demonstrate that CXCL5 recruits neutrophils in hepatocellular carcinoma to promote cancer growth and metastasis^10^. Zhang et al. indicate that tumor-infiltrating neutrophils are considered as an independent prognostic factor to predict overall survival in GC patients and could be incorporated into standard TNM staging system^40^. Increased tumor-infiltrating neutrophils is also associated with GC progression and reduced survival in patients^41^. Since CXCL5 activates neutrophils and induces the expression of pro-tumor factors in neutrophils, we determined whether CXCL5-activated neutrophils represented a pro-tumor phenotype. We found that the conditioned medium of CXCL5-activated neutrophils induced EMT in GC cells and enhanced their migration and invasion potential. The above-mentioned effects were also observed in neutrophils activated by TTCM in a CXCL5-dependent manner, suggesting that CXCL5 in GC microenvironment mediates neutrophil activation to promote GC metastasis. Our data are consistent with the others showing that overexpression of tumor-derived CXCL5 increases the number of intra- and peritumoral neutrophils and contributes to lymph node metastasis^24^.

Increasing studies suggest that tumor-associated neutrophils derived inflammatory factors play a key role in tumor progression. Cheng et al. demonstrate that neutrophils recruited into liver by cancer-associated fibroblasts orchestrate a pro-tumor function by suppressing T cell immunity through PD-L1 in hepatocellular carcinoma^42^. In breast cancer, the increased infiltration of neutrophils in lung promotes primary cancer cells disseminating to this site by generating GM-CSF and IL5^43^. In addition, tumor-associated neutrophils induce EMT to promote migration and invasion in gastric cancer cells by IL-17a^44^. We chose IL-6 and IL-23, two main factors that were increased in neutrophils when stimulated with CXCL5 for further study. The effects of CXCL5-activated neutrophils on promoting GC cell migration and invasion were impaired when IL-6 and IL-23 were blocked. Based on the above data, we propose that two mechanisms may be responsible for the tumor-promoting role of CXCL5 in GC. On one hand, CXCL5 promotes GC metastasis through activation of ERK signaling pathway and the upregulation of Snail in GC cells. On the other hand, CXCL5 recruits and activates neutrophils via activation of ERK and p38 signaling pathways, which then accelerates GC metastasis through the release of IL-6 and IL-23. Neutrophils are the main component of tumor microenvironment and play critical roles in tumor initiation, growth and metastasis^45^. Neutrophils form an inflammation-associated signaling network with HCC cells to drive cancer development. Tumor-activated neutrophils release BMP2 to trigger the expression of miR-301-3p in HCC cells, which down-regulates LSAMP and CYLD expression to increase the stemness of HCC cells. In turn, HCC cells produce higher levels of CXCL5 to recruit more neutrophils^46^. We showed that gastric cancer-derived CXCL5 activated neutrophils to promote GC metastasis, which implies a close link between CXCL5 and neutrophils in gastric cancer. However, the detailed mechanism and clinical correlation still needs further investigation.

The switch between EMT and its reverse process MET is responsible for primary cancer cell migration and invasion^47^ and contributes to the later stage of metastasis^48^. Chemokines function as essential regulatory factors in cancer metastasis by inducing EMT. CXCL5 induces EMT via multiple pathways in various cancers^14,22,31^. Chen et al. have revealed that macrophages recruited by tumor-derived CCL12 induce EMT program in tumor cells, which in turn enhance liver metastatic lesions^49^. In the current study, we showed an EMT phenotype in GC cells and a promotion of liver metastasis in GC after treatment with CXCL5 and conditioned medium of CXCL5-activated neutrophils. This is the first report showing that CXCL5 induces EMT and promotes liver metastasis of GC cells by modulating neutrophils, which suggests a diverse role of CXCL5 in GC progression.

**Fig. 7:**
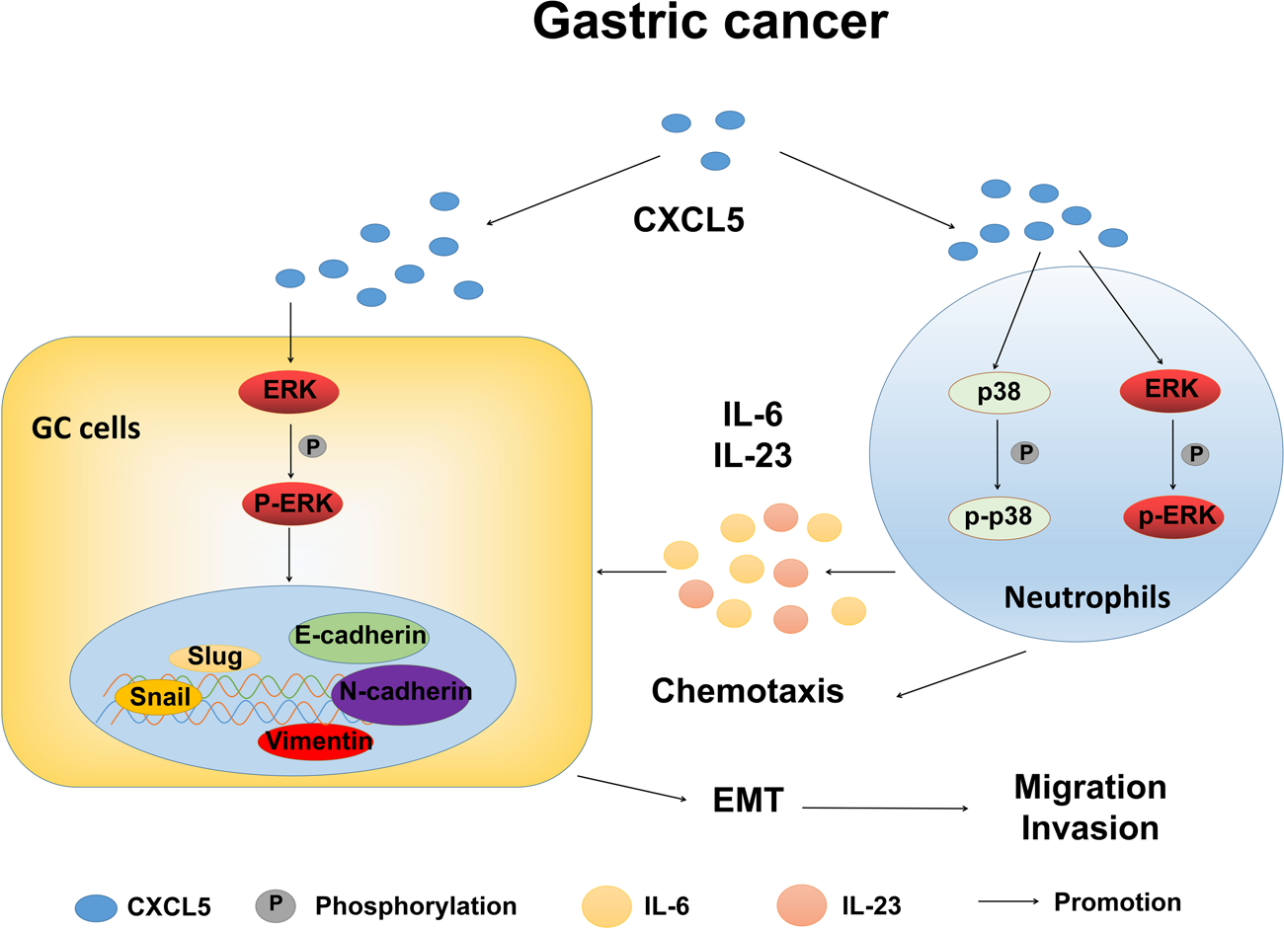
Proposed model for the role of CXCL5 in gastric cancer metastasis. High level of CXCL5 in tumor microenvironment promotes GC metastasis by enhancing GC cell migration and invasion through the induction of EMT. CXCL5 exerts these effects by two mechanisms: (1) CXCL5 directly activates ERK signaling pathway in GC cells to upregulate Snail expression; (2) CXCL5 induces pro-tumor activation of neutrophils via ERK and p38 signaling pathways, which leads to the release of increased levels of inflammatory factors (e.g, IL-6 and IL-23), subsequently enhancing the metastatic potential of gastric cancer cells.

In conclusion, we demonstrate in this study that high level of CXCL5 in tumor microenvironment promotes GC metastasis by enhancing GC cell migration and invasion through the induction of EMT. CXCL5 exerts these effects by activating ERK/Snail signaling pathway in GC cells and inducing pro-tumor activation of neutrophils (Fig. 7). Our study suggests that CXCL5 is a potential target for the treatment of gastric cancer metastasis.

## Materials and methods

### Patients and clinical samples

A total of 66 paired gastric cancer and adjacent noncancerous tissues (5 cm away from the tumor edge) were obtained from the Department of General Surgery, the Affiliated People’s Hospital of Jiangsu University, between April 2018 and May 2019. Written informed consent was obtained from all the patients, and this study was approved by the Institutional Ethical Committee of Jiangsu University. The patients included in this study had not received any preoperative therapies.

### Cell culture

Human normal gastric epithelial mucosa cell line (GES-1) and gastric cancer cell lines (HGC-27 and BGC-823) were purchased from the Institutes for Biological Sciences at the Chinese Academy of Sciences and were cultured in RPMI 1640 medium (Biological Industries) supplemented with 10% FBS (Life Technologies). All the cells were maintained at 37 °C in a humidified atmosphere of 5% CO_2_ incubator.

### Preparation of tumor tissue and normal tissue-derived conditioned medium

Fresh gastric tumor tissues and adjacent non-tumor tissues were cut into pieces about 1 cm^3^ and cultured in 1 mL serum-free RPMI 1640 medium in a humidified incubator with 5% CO_2_ at 37 °C for 24 h. The supernatants were then harvested and centrifuged at 3000 × *g* to remove cell debris. The culture supernatants from tumor tissues (TTCM) and normal tissues (NTCM) were stored at −80 °C refrigerator until use.

### Enzyme-linked immunosorbent assay

The concentrations of CXCL5 in tissue culture medium and the concentrations of IL-6 and IL-23 in neutrophil conditioned medium were measured by using an enzyme-linked immunosorbent assay (ELISA) kit according to the manufacturer’s instructions (Fcmacs Biotech, Nanjing, China).

### Isolation of human peripheral blood neutrophils

Human peripheral blood was collected from healthy donors and neutrophils were isolated by using Polymorphprep (Axis-Shield PoC AS), as previously described^19^ RBCs were lysed using hypotonic lysing procedure. Neutrophils were seeded in RPMI 1640 medium supplemented with 10% FBS.

### Stimulation of gastric cancer cells and neutrophils

Recombinant human CXCL5 (R&D Systems) was used to stimulate gastric cancer cells (HGC: 20 ng/mL, BGC: 60 ng/mL, 24 h) and neutrophils (100 ng/mL, 12 h). To generate conditioned medium, neutrophils from healthy donors were harvested and cultured in 40% TTCM or NTCM or stimulated with rhCXCL5 for 12 h. Afterward, neutrophils were changed to serum-free RPMI-1640 medium and cultured for 24 h. The conditioned medium from untreated neutrophils (NCM) and TTCM-treated neutrophil (TNCM) were harvested and centrifuged at 3000 × *g* to remove cell debries. Gastric cancer cells (2 × 10^5^) were incubated with 40% TNCM or NNCM for 24 h in 6-well plates. Specific ERK and p38 pathway inhibitors PD98059 and SB203580 were used at a concentration of 50 μM and 20 μM, respectively. For CXCL5 blockade study, CXCL5 neutralizing antibody (0.5 µg/mL) was used. For IL-6 and IL-23 blockade study, the working concentration of neutralizing antibodies was 2 µg/mL.

### Neutrophil chemotaxis assay

Neutrophils were seeded into the upper chamber (Corning; 4 µm pore size) at a density of 1 × 10^6^ in 100 µL serum-free medium. Recombinant human CXCL5 (rhCXCL5, 10, and 100 ng/mL) in RPMI 1640 medium was added to the lower wells. After incubation at 37 °C, 5% CO_2_ for 2 h, neutrophils that migrated to the lower chamber were collected and counted in Neubauer chambers. Neutrophils migrated toward RPMI 1640 medium alone was used as the negative control.

### Cell migration and invasion assays

For migration assay, the cells (2 × 10^4^) were collected and seeded into the upper chamber (8 µm) in 24-well plates (Corning). For invasion assay, the diluted (1:3) basement Matrigel was added into each chamber and let to polymerize at 37 °C for 30 min. The cells (1 × 10^5^) were seeded into the upper chamber. The lower chamber was filled with 600 µL RPMI 1640 medium supplemented with 10% FBS. After incubation for 24 h, the migrated or invaded cells on the bottom of the insert were fixed, stained, and photographed under the microscope at ×20 magnification. Five fields were randomly selected for quantification. All the experiments were performed in triplicates.

### Real-time quantitative PCR

Total RNA was extracted from tissues and cells using TRIzol reagent (Invitrogen) according to the manufacturer’s protocol. cDNAs were synthesized from total RNA (1 µg) by using HiScript First Strand cDNA Synthesis Kit (Vazyme Biotech). QRT‐PCR was carried out on a Bio-Rad CFX96 detection system (Bio-Rad) by using SYBR Green I real-time detection kit (Cwbio). The forward and reverse primer sequences are listed in Table 2. Gene expression was determined by the 2^−ΔΔCt^ method using β-actin as an internal control. All the experiments were repeated for at least three times.

**Table 2:** The sequences of primers for target genes.

Target	Sequence (5′-3′)	Size (bp)	Tm (^o^C)
E-cadherin	F:5′-CGCATTGCCACATACACTCT-3′	252	58
	R:5′-TTGGCTGAGGATGGTGTAAG-3′		
N-cadherin	F:5′-AGTCAACTGCAACCGTGTCT-3′	337	58
	R:5′-AGCGTTCCTGTTCCACTCAT-3′		
Slug	F:5′-CCTGGTTGCTTCAAGGACAC-3′	395	58
	R:5′-TCCATGCTCTTGCAGCTCTC-3′		
Snail	F:5′-GCGAGCTGCAGGACTCTAAT-3′	310	58
	R:5′-GCCTCCAAGGAAGAGACTGA-3′		
Vimentin	F:5′-GGACCTCTACGAGGAGGAGAT-3′	207	58
	F:5′-GCCAGAGACGCATTGTCAAC-3′		
Actin	F: 5′-CACGAAACTACCTTCAACTCC-3′	265	58
	R: 5′-CATACTCCTGCTTGCTGATC-3′		
IL-6	F: 5′-TACATCCTCGACGGCATCTC-3′	252	60
	R: 5′-AGCTCTGGCTTGTTCCTCAC-3′		
IL-8	F: 5′-GCTCTGTGTGAAGGTGCAGTTT-3′	144	60
	R: 5′-TTCTGTGTTGGCGCAGTGT-3′		
IL-17	F: 5′-GCCATAGTGAAGGCAGGAA-3′	163	60
	R: 5′-GTGAGGTGGATCGGTTGTAG-3′		
IL-23	F: 5′-CTCCCTGATAGCCCTGTGG-3′	145	60
	R: 5′-TGAAGCGGAGAAGGAGACG-3′		
PD-L1	F: 5′-TGGCATTTGCTGAACGCATTT-3′	120	60
	R: 5′-TGCAGCCAGGTCTAATTGTTTT-3′		
MMP-9	F: 5′-ACGTCTTCCAGTACCGAGAG-3′	126	60
	R: 5′-GGCACTGCAGGATGTCATAG-3′		
TNF-α	F: 5′-CCGAGTGACAAGCCTGTAGC-3′	260	60
	R: 5′-AGGAGGTTGACCTTGGTCTG-3′		
TGF-β	F: 5′-AGAGTGCCTGAACAACGGATT-3′	117	60
	R: 5′-CCATTCGCCTTCTGCTCTT-3′		
VEGF	F: 5′-AAAGGGTGGAGGTGACTG-3′	134	60
	R: 5′-GACATAAATGACCGAGGC-3′		
CXCL5	F: 5′-GACGGTGGAAACAAGGAAAA-3′	218	58
	R: 5′-GCTTAAGCGGCAAACATAGG-3′		

### Western blot

Total proteins were separated on a 10% SDS‐PAGE gel and transferred onto a polyvinylidene difluoride membrane (Millipore). Then, the membrane was blocked by TBST containing 5% non–fat milk for 1 h and incubated overnight at 4 °C with the primary antibodies against ERK1/2, p-ERK1/2, p38, p-p38, E-cadherin, N-cadherin, Vimentin, Snail, Slug (1:1000; Cell Signaling Technology). GAPDH (1:5000; Proteintech) was used as internal control. Afterward, the blots were labeled for 1 h with HRP-conjugated secondary antibody (1:3000; Invitrogen Life Technologies). Finally, the blots were visualized in ChemiDoc XRS system (Bio‐Rad).

### Immunohistochemistry

Formalin-fixed and paraffin-embedded liver metastatic nodules were sectioned and used for IHC Staining. Antigen retrieval was performed using a pressure cooker for 30 min in antigen unmasking solution (ETDA, pH 8.0), followed by incubation in 3% H_2_O_2_ for 30 min and blocked with PBS containing 5% BSA for 30 min. Afterward, the tissue sections were incubated with primary antibodies against human E-cadherin (1:200; Cell Signaling Technology) overnight at 4 °C, followed by a HRP-conjugated secondary antibody (Boster) for 1 h at room temperature. Then, immunodetection was performed by using DAB (Maxim) for 2 min and the sections were counterstained with hematoxylin for 30 s. Finally, images were obtained by TE2000 microscope (Nikon). The proportion of positive area was calculated with ImageJ software.

### Animal study

Male BALB/c nude mice (21–25 g weight; aged 4–6 weeks) were purchased from the Model Animal Research Center of Nanjing University and maintained in specific pathogen-free (SPF) conditions in accordance with the institutional policies. The mice received sterile rodent chow and water ad libitum and were housed in sterile filter-top cages with 12-h light/dark cycles. RPMI-1640, rhCXCL5, 0-NCM (conditioned medium from non-primed neutrophils), 100-NCM (conditioned medium from rhCXCL5-primed neutrophils) treated BGC-823 cells were collected in PBS and intraperitoneally injected into the mice (2 × 10^6^ cells/mice, *n* = 5). The mice were regularly fed. At 3 weeks after injection, the mice were sacrificed, the abdominal colonized tumors were observed and the number of metastatic nodules in the livers were counted. The protocol was approved by the Animal Use and Care Committee of Jiangsu University.

### Statistical analysis

All the results were expressed as mean ± SD. Statistical analyses were performed using Student’s *t*-test with GraphPad Prism Version 5.0 software (GraphPad Software, La Jolla, CA, USA). *P* < 0.05 was considered to be statistically significant.

## Data Availability

All of the data and material in this paper are available when requested.
